# Spatial distribution and predictors of lifetime experience of intimate partner violence among women in South Africa

**DOI:** 10.1371/journal.pgph.0000920

**Published:** 2023-01-24

**Authors:** Obasanjo Afolabi Bolarinwa, Zemenu Tadesse Tessema, Joshua Okyere, Bright Opoku Ahinkorah, Abdul-Aziz Seidu

**Affiliations:** 1 Department of Public Health Medicine, School of Nursing and Public Health, University of KwaZulu-Natal, Durban, South Africa; 2 Institute for Advanced Studies in the Humanities, University of Edinburgh, Hope Park Square, Edinburgh, United Kingdom; 3 Department of Epidemiology and Biostatistics, Institute of Public Health, College of Medicine and Health Sciences, University of Gondar, Gondar, Ethiopia; 4 Department of Epidemiology and Preventive Medicine, School of Public Health and Preventive Medicine, Monash University, Melbourne, Australia; 5 Department of Population and Health, University of Cape Coast, Cape Coast, Ghana; 6 School of Public Health, University of Technology Sydney, Sydney, Australia; 7 Centre for Gender and Advocacy, Takoradi Technical University, Takoradi, Ghana; 8 College of Public Health, Medical and Veterinary Sciences, James Cook University, Townsville, Australia; University of Portsmouth, UNITED KINGDOM

## Abstract

In recent times, intimate partner has gained significant attention. However, there is limited evidence on the spatial distribution and predictors of intimate partner violence. Therefore, this study examined the spatial distribution and predictors of intimate partner violence in South Africa. The dataset for this study was obtained from a cross-sectional survey of the 2016 South Africa Demographic and Health Survey. We adopted both spatial and multilevel analyses to show the distribution and predictors of intimate partner violence among 2,410 women of reproductive age who had ever experienced intimate partner violence in their lifetime in South Africa. The spatial distribution of intimate partner violence in South Africa ranged from 0 to 100 percent. Western Cape, Free State, and Eastern Cape were predicted areas that showed a high proportion of intimate partner violence in South Africa. The likelihood of experiencing intimate partner violence among women in South Africa was high among those who were cohabiting [aOR = 1.41; 95%(CI = 1.10–1.81)] and women who were previously married [aOR = 2.09; 95%(CI = 1.30–3.36)], compared to women who were currently married. Women who lived in households with middle [aOR = 0.67; 95%(CI = 0.48–0.95)] and richest wealth index [aOR = 0.57; 95%(CI = 0.34–0.97)] were less likely to experience lifetime intimate partner violence compared to those of the poorest wealth index. The study concludes that there is a regional variation in the distribution of intimate partner violence in South Africa. A high prevalence of intimate partner violence was found among women who live in the Western Cape, Free State, and Eastern Cape. Furthermore, predictors such as women within the poorest wealth index, women who were cohabiting and those who were previously married should be considered in the development and implementation of interventions against intimate partner violence in South Africa.

## Background

In recent years, violence perpetrated by an intimate partner has received a lot of attention [1]. The World Health Organization (WHO) [2] defines intimate partner violence (IPV) as encapsulating acts of violence, physical aggression, psychological abuse, controlling behaviors, and sexual coercion that is inflicted by former or current spouse or another intimate partner. IPV may manifest as emotional, physical, and sexual violence. Available evidence indicates that one-third of women globally have ever experienced IPV in their lifetime [2]. Within the southern region of Africa, it is also estimated that nearly 30% of women have ever experienced IPV in their lifetime [3].

Evidence from the 2017 Statistics South Africa report shows that approximately one out of five adult women in South Africa had ever experienced violence by a partner [4]. Also, evidence from the 2016 South African Demographic and Health Survey (SADHS) indicates that the prevalence of IPV among women was 21.9% which is lower than the regional IPV prevalence [5]. This variation between the IPV prevalence as reported in the 2016 SADHS and the Southern Africa’s IPV prevalence could be attributable to spatial variations. Hence, necessitating the urgency for an investigation into the spatial distribution and predictors of IPV.

The imperativeness to find lasting solutions to IPV is emphasised by its associated adverse effects [6]. IPV has been found to have adverse effects on the physical health of women (e.g., injuries) [5]. It is also associated with many mental health issues, including anxiety, depression, suicidal ideation and attempt, and posttraumatic stress disorders [7, 8]. Moreover, IPV that manifests in the form of sexual abuse tends to be a conduit for perpetuating unintended pregnancies, which in most cases result in adverse sexual and reproductive health outcomes such as unsafe abortions and facilitate the spread of sexually transmitted infections [9–11]. This makes IPV a serious public health concern that affects several millions of women worldwide [2, 12].

Previous studies conducted in South Africa indicate that IPV is associated with several factors such as age [13], multiple sexual partnerships [14], childhood experience of abuse [15], as well as engagement in transactional sex and health inequalities [14, 16]. Beyond these associated factors, evidence from recent studies conducted in Ethiopia [5, 17], Ghana [18], Uganda [19], Nigeria [6] and Afghanistan [8] suggest that there are spatial variations in the distribution of IPV across the respective countries that have been minimally explored in extant literature.

Consequently, studies in South Africa have not investigated the spatial distribution of IPV, thereby creating a literature gap that this study seeks to address. Therefore, we aimed to examine the spatial distribution and predictors of IPV in South Africa. The potential findings of this study are useful for South Africa’s journey towards the realisation of the Sustainable Development Goals (SDGs), particularly Goal 5, which seeks to achieve gender equality and empower all women and girls by 2030 [20], and also in formulating policies and interventions that will reduce regional spatial variation in IPV prevalence in South Africa.

## Methods and materials

### Data source

The dataset for this study was obtained from the most recent cross-sectional survey of the 2016 SADHS. The SADHS is a nationally representative survey used to examine socio-demographic, health, and other health-related indicators such as intimate partner violence [21]. Following a two-stage stratified sampling technique, the 2016 SADHS used a probability proportional to size sampling of Primary Sampling Units (PSUs) at the first stage to sample respondents, and this was followed by a systematic sampling of dwelling units. For the 2016 SADHS, a total of 15,292 households, out of which 13, 288 were occupied, were selected for the sample. A total of 8,514 women were initially identified for the domestic violence module in the 2016 SADHS. However, only 4,357 women were selected and interviewed. From this number, 2410 reproductive-aged women with complete information on IPV and all the variables of interest in this study were included. The sampling, pretesting, and general methodology of the 2016 SADHS have been published elsewhere [22]. We followed the strengthening of the reporting of observational studies in Epidemiology in writing this manuscript [23]. The dataset is freely accessible for download via https://dhsprogram.com/methodology/survey/survey-display-390.cfm

### Variables

#### Dependent variable

The dependent variable was lifetime experience of IPV. Lifetime experience of IPV was generated from three key variables (sexual violence, emotional violence, and physical violence). These variables were derived from several questions in the domestic violence module related to the number of violent acts experienced by a woman. On physical violence, each respondent was asked whether her last partner ever pushed her; shook or threw something at her; slapped her; punched her with his fist or something harmful; kicked or dragged her; strangled or burnt her; or threatened her with a knife, gun or other weapons; and twisted her arm or pulled her hair. Questions on emotional violence focused on whether a respondent’s last partner ever: humiliated her, threatened to harm her; and insulted or made her feel bad. On sexual violence, respondents were asked whether the partner ever physically forced the respondent into unwanted sex; whether the partner ever forced her into other unwanted sexual acts; and whether the respondent has been physically forced to perform sexual acts she did not want to. Details of the questions for each element of IPV can be found in previous studies [24, 25]. Responses for each question were ‘yes’ and ‘no’. A respondent who had experienced at least one of the violent acts was considered as ever experienced physical, emotional, or sexual violence. From the questions asked on the experience of physical, emotional, and sexual violence, IPV was generated, with respondents experiencing at least one of these violent acts regarded as ever had IPV and otherwise [26–28].

#### Independent variables

Individual and contextual-level (household and community) factors were considered as independent variables in this study. The individual-level factors included the age of respondents (15–24, 25–34, and 35+), educational level (No education, primary, secondary/higher), husband/partner’s educational level (No education, primary, and secondary/higher), marital status (currently married, cohabiting, and previously married), working status (not working and working), ethnicity (Black, White, Coloured & others), parity (0, 1–3, and 4+), Media exposure (yes and no). The contextual-level factors were place of residence (urban and rural), wealth index (poorest, poorer, middle, richer, and richest), region (Western Cape, Eastern Cape, Northern Cape, Free state, KwaZulu-Natal, Northwest, Gauteng, Mpumalanga, and Limpopo), sex of household head (male and female), community literacy level was the proportion of women who can read and write (low and medium), and Community socioeconomic status was the proportion of women in the richest household quintile (low and high). Both community literacy level and community socio-economic status were derived from clusters using principal component analysis. All these variables were considered based on their theoretical and practical relevance to IPV and their availability in the 2016 SADHS dataset [26–29].

Bronfenbrenner’s ecological theory guided the selection of the explanatory variables included in this study [29]. The theory emphasised that the actions perpetrated by an individual are results of the exposure they have had at individual, family, neighborhood, or community levels. These actions result in adverse health outcomes, including violence [29, 30]. Thus, this current study extracted variables based on individual and contextual levels to predict the factors associated with IPV among women of reproductive age in South Africa.

### Statistical analyses

The data were analysed using both spatial and multilevel analysis.

### Spatial analysis

The weighted frequency of IPV with cluster number and geographic coordinate data was merged using Stata 16 software. A total of 746 enumeration areas or clusters were identified for the 2016 SADHS. From these clusters, 10 clusters had no location measurement. A total of 736 clusters were included in the final analysis. The cleaned dataset was exported to excel and then imported to ArcGIS 10.7 for mapping.

### Spatial autocorrelation

The spatial autocorrelation (Global Moran’s I) statistics measure was used to check whether IPV was dispersed, clustered, or randomly distributed. The Moran’s I closer to +1 indicates IPV clustered in a certain area in South Africa, I closer to -1 shows the IPV is dispersed, and I closer to 0 indicates IPV is distributed randomly in South Africa.

### Spatial interpolation

It is very expensive and laborious to collect reliable data in all areas of the country to know the burden of IPV in South Africa. Taking the sample enumeration areas and predicting unsampled areas is one technique of prediction of IPV in South Africa. The ordinary Kriging spatial interpolation method was used to predict IPV in unobserved areas of South Africa based on Enumeration areas in which a sample has been taken.

### Multilevel analysis

A multilevel logistic regression models were fitted to evaluate the individual and contextual-level factors linked to IPV. The choice of multilevel logistic regression to examine the predictors of IPV in South Africa was because of its unique features in showing the results of both individual and contextual factors separately, and the analysis showed cluster variation in IPV in South Africa [31, 32]. Consequently, women were nested within households, and households were nested within clusters. Clusters were considered as a random effect to account for the unexplained variability at the contextual level. Four models were fitted, made up of the empty model (Model I), which contained no predictors (random intercept). Following that, model II included only individual-level variables, model III included only contextual-level variables, and model IV included both individual-level and contextual-level variables. The odds ratio and related 95% confidence intervals were provided for all models. These models were fitted by a Stata command “melogit” to identify the independent variables that are associated with IPV in South Africa [31].

The log-likelihood ratio (LLR), Akaike Information Criteria (AIC) measure, and Schwarz’s Bayesian Information Criteria (BIC) were used to compare models. All three were used to assess model fitness. They complement the strengths and weaknesses of one another during the comparisons and are very useful. The model with the highest LLR and BIC and the lowest AIC is considered the best fit model. The multicollinearity test, which used the variance inflation factor (VIF), revealed no evidence of collinearity among the independent variables. The women population sample weight (v005/1,000,000) was used in all analyses to account for over-and under-sampling, while the svy command was used to account for the complex survey design and generalizability of the results [32]. All the analyses were carried out using Stata version 16 sofrware.

### Ethical approval

Since the authors of this manuscript did not collect the data, we sought permission from the MEASURE DHS website and access to the data was provided after our intent for the request was assessed and approved on the 10th of January, 2021.

## Results

### Socio-demographic characteristics of respondents

A total of 2,410 women were included in the study. At the individual level, 1,248 (51.78%) of the respondents were aged 35 and above, 2,091 (86.74%) of women had secondary/higher education, 1,737 (72.06%) of husbands/partners had secondary/higher education, 1,359 (56.38%) were currently married. Again, 1,997 (82.87%) of women’s ethnicity were Black, and 2,223 (92.24%) were exposed to mass media. At the contextual level, 1,737 (72.07%) of women resided in the urban areas, 525 (21.79%) were from a middle household, 774 (32.10%) were residing in Gauteng, 1,590 (65.96%) were from a community with medium literacy level, and 1,511 (62.70%) were from a community with low socioeconomic status (Table 1).

**Table 1:** Individual & household-level characteristics of respondents.

Individual-level n = 2,410	Weighted Frequency	Weighted Percentage
**Age of respondent**		
15–24	223	9.25
25–34	939	38.97
35 & above	1,248	51.78
**Women’s level of education**		
No Education	72	2.97
Primary	248	10.29
Secondary/higher	2,091	86.74
**Husband/Partner’s level of education**		
No Education	432	17.92
Primary	242	10.02
Secondary/higher	1,737	72.06
**Marital status**		
Currently Married	1,359	56.38
Cohabitating	768	31.85
Previously Married	284	11.77
**Working status**		
Not working	1,332	55.27
Working	1,078	44.73
**Ethnicity**		
Black	1,997	82.87
White	131	5.43
Coloured & Others	282	11.71
Parity		
0	208	8.63
1–3	1,828	75.85
4 & above	374	15.52
**Exposure to media**		
No	187	7.76
Yes	2,223	92.24
Household-level		
**Place of residence**		
Urban	1,737	72.07
Rural	673	27.93
**Wealth index**		
Poorest	427	17.72
Poorer	517	21.47
Middle	525	21.79
Richer	452	18.76
Richest	489	20.27
**Region**		
Western cape	334	13.85
Eastern Cape	240	9.96
Northern Cape	58	2.40
Free state	130	5.38
KwaZulu-Natal	248	10.29
Northwest	177	7.33
Gauteng	774	32.10
Mpumalanga	204	8.46
Limpopo	247	10.23
**Sex of household head**		
Male	1,734	71.95
Female	676	28.05
**Community literacy level**		
Low	820	34.04
Medium	1590	65.96
**Community socioeconomic status**		
Low	1,511	62.70
High	899	37.30

SADHS, 2016

### Spatial analysis result

#### Spatial distribution of Intimate partner violence in South Africa

**Fig 1:**
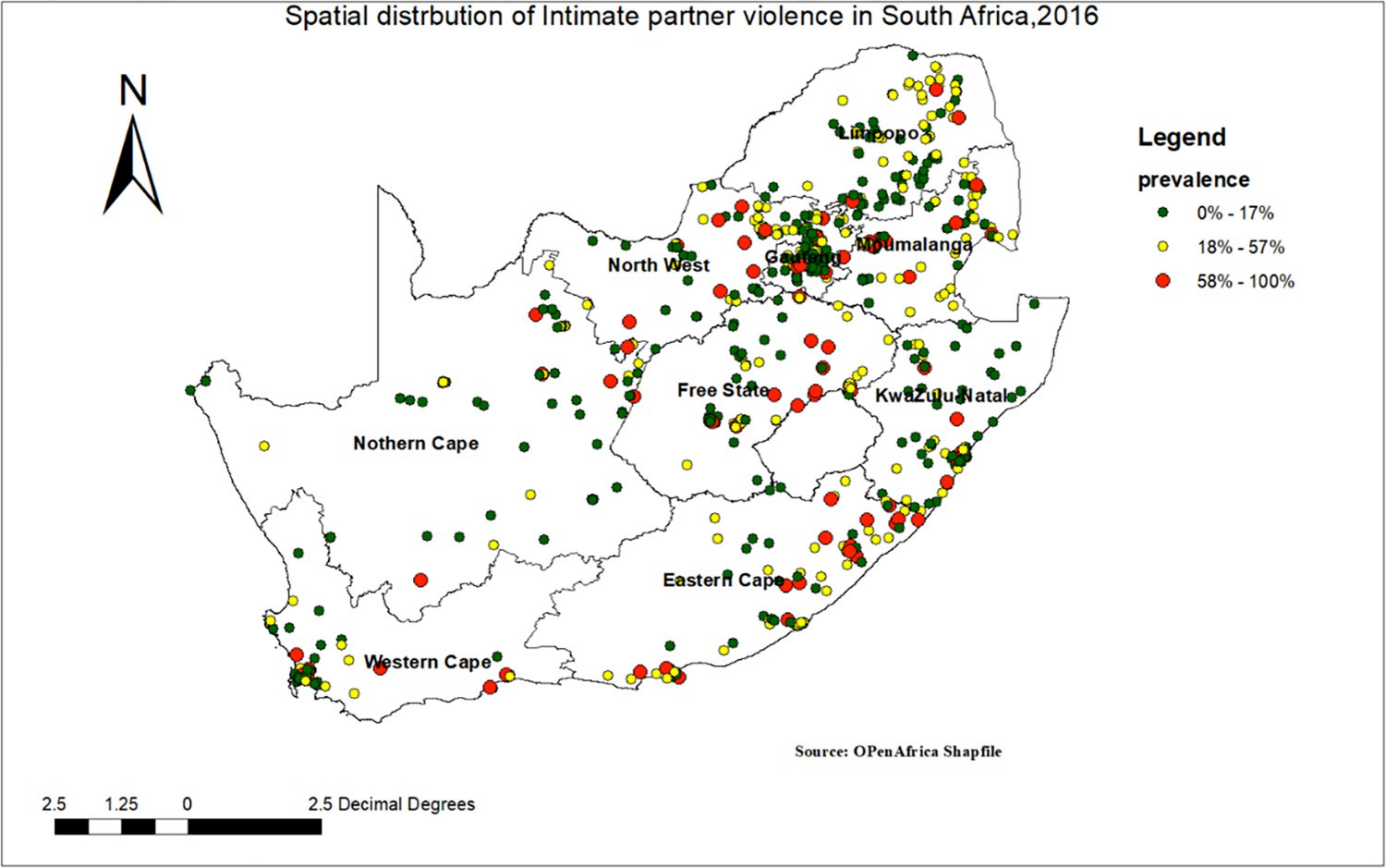
Spatial distribution of IPV in South Africa, 2016. Attributions to ESRI.

The spatial distribution of IPV in South Africa ranged from 0 to 100 percent. As the spatial distribution map of IPV revealed, the red colour indicates a high IPV percentage, ranging from 81% to 100%. The yellow colour indicates the proportion of IPV ranges from 41% to 80%, and the blue colour indicates the proportion of IPV from 0 to 40% (**Fig 1**).

#### Spatial autocorrelation of IPV in South Africa

**Fig 2:**
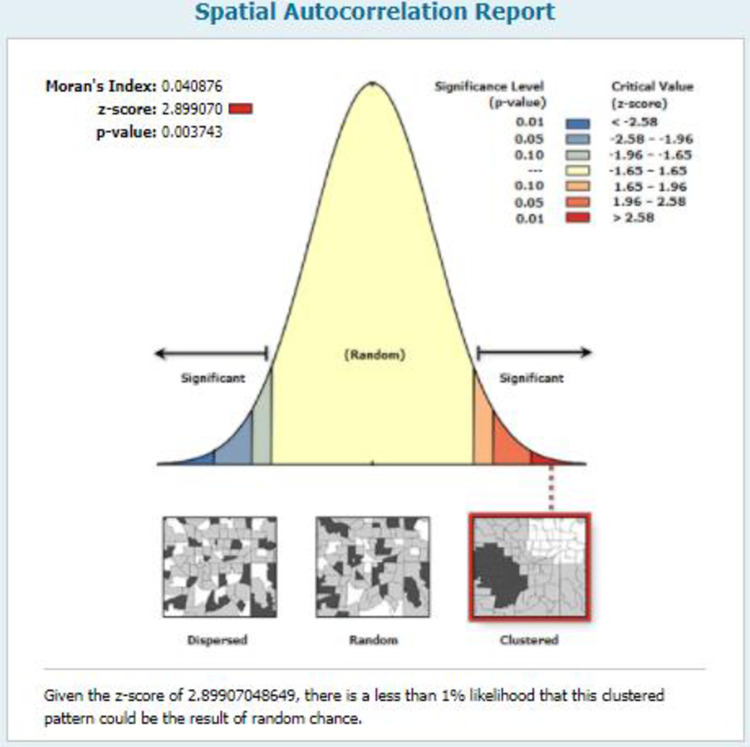
The spatial autocorrelation of IPV in South Africa, 2016.

The spatial autocorrelation result shows whether IPV in South Africa is dispersed, clustered, or random. The finding of the spatial autocorrelation analysis result revealed that there is a clustering effect in IPV across in South Africa. The clustered patterns (on the right-side red box) showed a clustering effect on IPV in South Africa. The outputs automatically generated keys on each panel’s right and left sides. Given the z-score of 2.89 (p-value = <0.003), there is an indication that there is less than a 1% likelihood that this clustered pattern could be the result of random chance. The bright red and blue colours of the end tails indicate an increased significance level (**Fig 2**).

#### Spatial interpolation or prediction

**Fig 3:**
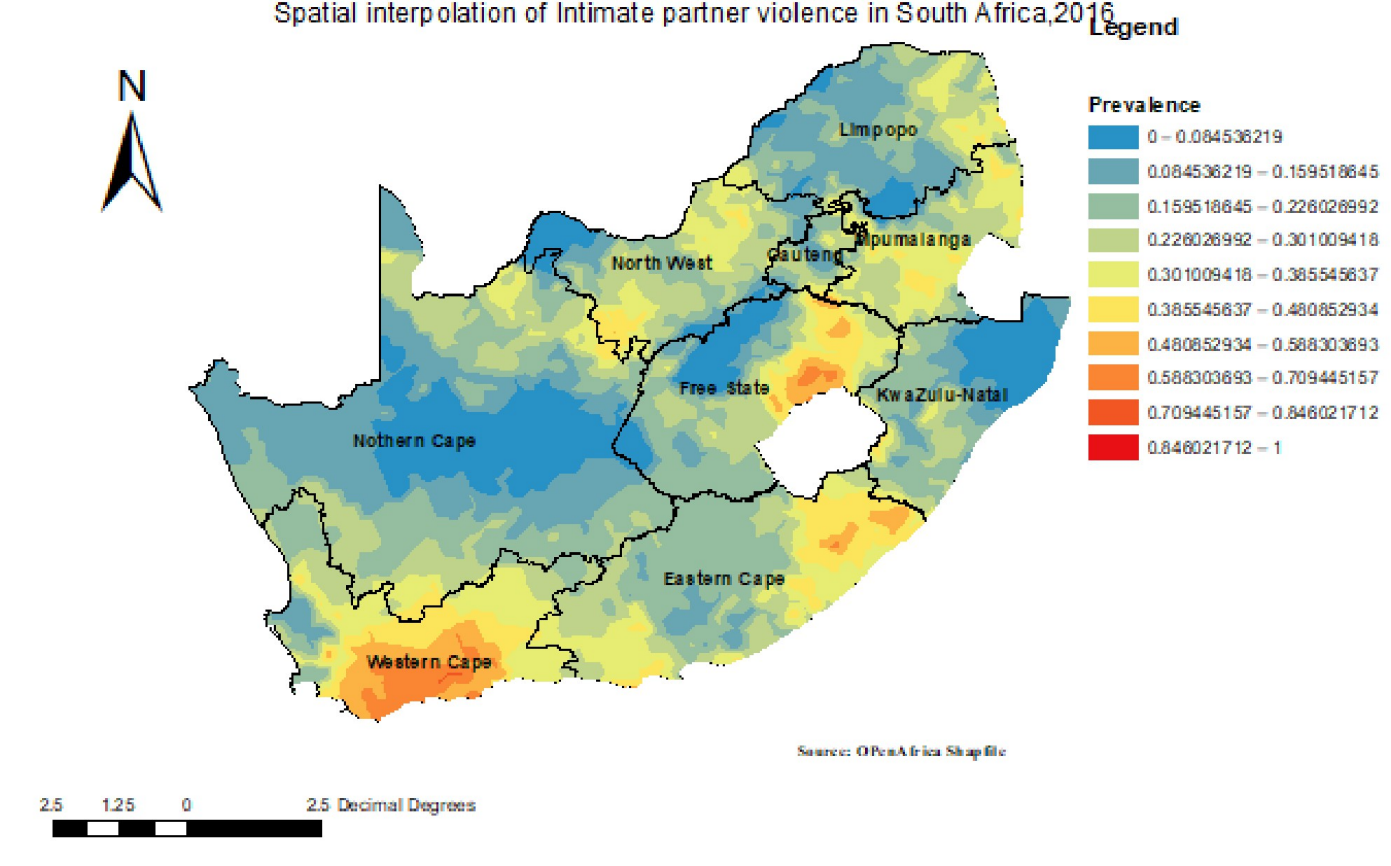
The interpolation of intimate partner violence in South Africa, 2016. Attributions to ESRI.

The spatial interpolation technique shows the predicted proportion of IPV for unsampled areas based on the sampled area. The ordinary Kriging interpolation method of the analysis indicated that IPV in South Africa ranges from 0% to 100%. Western Cape, Free State, and Eastern Cape were predicted areas that showed a high proportion of IPV in South Africa (**Fig 3**).

#### Fixed effects (measures of associations) results

At the individual-level factors, the likelihood of experiencing IPV among women in South Africa was high among those who were cohabiting [aOR = 1.41; 95%(CI = 1.10–1.81)] and women who were previously married [aOR = 2.09; 95%(CI = 1.30–3.36)], compared to women who were currently married. At the household/community level, women who were from the middle wealth index household [aOR = 0.67; 95%(CI = 0.48–0.95)], those women from richest wealth index household [aOR = 0.57; 95%(CI = 0.34–0.97)], women residing in Northern Cape province [aOR = 0.47; 95%(CI = 0.28–0.78)], KwaZulu-Natal province [aOR = 0.42; 95%(CI = 0.24–0.75)], North West province [aOR = 0.57; 95%(CI = 0.34–0.98)], Gauteng province [aOR = 0.47; 95%(CI = 0.27–0.80)], and Limpopo province [aOR = 0.50; 95%(CI = 0.29–0.85)] were less likely to experience intimate sexual violence compared to women from poorest wealth index household and those residing in Western Cape.

#### Random effects (measures of variations) results

The empty model (Model 0), as shown below in Table 2, depicted a substantial variation in the likelihood of IPV among women in South Africa across the Primary Sampling Units (PSUs) clustering [σ2 = 0.43; 95%(CI = 0.25–1.66)]. The Model 0 indicated that 11% of the variation in IPV among women in South Africa was attributed to the variation in Intra-Class Correlation, i.e. (ICC = 0.11). The variation between-cluster decreased to 10% (0.10) in Model I (individual level only). In the contextual-level only (Model II), the ICC decreased further to 6%, while the ICC declined to a 5% incomplete model with both the individual and contextual-level factors (Model III). This further reiterates that the likelihood of IPV in South Africa is attributed to the clustering variation in PSUs. Moreover, the model with the highest LLR and the lowest AIC (Model III) is considered the best fit. Therefore, Model III, the complete model with the selected individual and contextual-level factors, was selected to predict the likelihood of IPV among women in South Africa.

**Table 2:** Multilevel logistic regression models for individual and contextual level predictors of intimate partner violence in South Africa.

Variables	Model 0	Model I	Model II	Model III
		aOR[95% CI]	aOR[95% CI]	aOR[95% CI]
**Fixed effects results**				
**Individual-level variables**				
**Age of respondent**				
15–24		RC		RC
25–34		0.73 [0.50–1.05]		0.76 [0.52–1.11]
35 & above		0.64*[0.43–0.95]		0.70 [0.47–1.03]
**Women’s educational level**				
No Education		RC		RC
Primary		1.75 [0.94–3.24]		1.62 [0.88–3.00]
Secondary & above		1.42 [0.79–2.57]		1.40 [0.77–2.54]
**Spouse educational level**				
No Education		RC		RC
Primary		0.85 [0.53–1.36]		0.83 [0.52–1.32]
Secondary & above		0.80 [0.54–1.20]		0.85 [0.57–1.27]
**Marital status**				
Currently Married		RC		RC
Cohabitating		1.43**[1.2–1.83]		1.41**[1.10–1.81]
Previously Married		2.03**[1.28–3.20]		2.09**[1.30–3.36]
**Working status**				
No		RC		RC
Yes		0.97 [0.78–1.20]		0.97 [0.78–1.20]
**Ethnicity**				
Black		RC		RC
White		0.45*[0.23–0.88]		0.52 [0.26–1.03]
Coloured & others		0.97 [0.70–1.34]		0.99 [0.66–1.50]
**Parity**				
0		RC		RC
1–3		1.03 [0.70–1.51]		1.06 [0.73–1.55]
4 & above		1.19 [0.75–1.87]		1.13 [0.72–1.79]
**Exposure to media**				
No		RC		RC
Yes		1.03 [0.71–1.50]		1.18 [0.79–1.76]
**Contextual-level variables**				
**Place of residence**				
Urban			RC	RC
Rural			0.95 [0.82–1.09]	0.98 [0.74–1.30]
**Wealth index**				
Poorest			RC	RC
Poorer			0.96 [0.71–1.30]	0.92 [0.67–1.27]
Middle			0.66*[0.48–0.91]	0.67*[0.48–0.95]
Richer			0.62*[0.43–0.90]	0.72 [0.49–1.06]
Richest			0.39***[0.24–0.64]	0.57*[0.34–0.97]
**Region**				
Western cape			RC	RC
Eastern Cape			1.28 [0.80–2.03]	1.29 [0.79–2.11]
Northern Cape			0.51**[0.31–0.84]	0.47**[0.28–0.78]
Free state			1.03 [0.31–0.84]	0.95 [0.57–1.59]
KwaZulu-Natal			0.41**[0.24–0.71]	0.42**[0.24–0.75]
Northwest			0.64 [0.39–1.05]	0.57*[0.34–0.98]
Gauteng			0.49**[0.30–0.79]	0.47**[0.27–0.80]
Mpumalanga			0.89 [0.56–1.42]	0.79 [0.47–1.33]
Limpopo			0.48**[0.29–0.80]	0.50*[0.29–0.85]
**Head of household**				
Male			RC	RC
Female			1.19 [0.96–1.48]	0.90 [0.70–1.16]
**Community literacy level**				
Low			RC	RC
Medium			0.89 [0.70–1.12]	0.90 [0.71–1.15]
**Community socioeconomic status**				
Low			RC	RC
High			1.07 [0.78–1.48]	1.01 [0.73–1.40]
**Random effects results**				
PSU Variance (95% CI)	0.43[0.24–0.80]	0.54[0.46–0.66]	0.23[0.08–0.63]	0.42[0.33–0.52]
ICC	0.12	0.10	0.06	0.03
LR Test	χ2 = 17.97,p<0.001	χ2 = 13.30, p<0.001	χ2 = 5.01, p<0.05	χ2 = 3.70, p<0.05
Wald χ2	Reference	59.19***	76.12***	112.56***
**Model fitness**				
Log-likelihood	-1304.21	-1273.36	-1264.85	-1244.36
AIC	2612.42	2578.72	2565.69	2552.72
Number of clusters	661	661	661	661

Weighted SADHS, 2016

Exponentiated coefficients; 95% confidence intervals in brackets; AOR = adjusted Odds Ratios; CI = Confidence Interval; RC = Reference Category.

*p< 0.05

**p< 0.01

***p< 0.001.

PSU = Primary Sampling Unit; ICC = Intra-Class Correlation; LR Test = Likelihood ratio Test; AIC = Akaike’s Information Criterion

Model 0 is the null model, a baseline model without any independent variable.

Model, I is adjusted for individual-level variables (Age of respondent, women’s educational level, spouse’s educational level, marital status, currently working, ethnicity, parity, and media exposure).

Model II is adjusted for contextual-level variables (Place of residence, wealth index, region, sex of household head, community literacy level, and community socioeconomic status).

Model III is the final model adjusted for individual and contextual-level variables

## Discussion

We examined the spatial distribution and predictors of lifetime experience of IPV in South Africa. Our findings indicate that IPV varied within South Africa; Western Cape, Free State, and Eastern Cape were the significant hotspots for IPV. This finding showed that there is spatial variation in the distribution of IPV within South Africa. This is in line with existing evidence from Ghana [18], Uganda [19] and Ethiopia [5]. A plausible justification in support of our findings could be the pervasiveness of misconceptions about IPV and prevailing cultural beliefs that reinforce acts that fall under the category of IPV [33]. Thus, emphasizing the need to target communities that are hotspots of IPV to realize the successful implementation of policies and programs that aim to eliminate IPV in all forms.

The multilevel analysis results show that marital status, wealth index, and region were the significant predictors of IPV in South Africa. We found a decreasing likelihood of IPV in relation to a higher wealth index. Thus, the odds of experiencing IPV among women from the richest household were significantly lower compared to those from the poorest households. This is supported by related studies conducted in Ethiopia [34] and Nepal [35]. Moreover, women from poor households tend to depend heavily on their partners for economic support [36]; as such, the women themselves become tolerable and normative violence from intimate partners, thereby exacerbating the risk of experiencing IPV [37].

Marital status was also found to be a significant predictor of IPV in South Africa. Contrary to earlier studies, Vatnar and Bjørkly [38] argue that patriarchal domination, sexual jealousy, and possessiveness exacerbate the risk of IPV among married women. We observed that cohabiting women and those previously married were at higher risk of experiencing violence from an intimate partner. The reason why the marital status was the only associated predictor of IPV in South Africa at the individual level could be that the selected variables cannot act on alone except it is exacerbated by community factors such as contextual or cultural perspectives that are beyond the control of the victim [6, 10, 39]. The result is consistent with previous studies that have reported an elevated likelihood of experiencing IPV among separated and divorced women to be 30 times and 9 times higher, respectively, compared to married women [40]. According to Brownridge [40], men attempt to use violence as a conduit to reclaim their dominance and rights over their former spouse or partner. Hence, this may explain the high risk of experiencing IPV in previously married women compared to cohabiting women and married women.

Lastly, our study reveals that women from Limpopo, Gauteng, Mpumalanga, and North West provinces had lower odds of experiencing IPV. Our finding aligns with Leddy, and Lippman [41], that found the likelihood of experiencing physical IPV lower within the Mpumalanga province. The authors linked this low incidence of IPV in the Mpumalanga province to the community’s collective efficacy (i.e., communal trust and solidarity among community members and preparedness to arbitrate on behalf of the common good), which serves as a protective factor against IPV among women [41]. This may explain the lower likelihood of women in the aforementioned regions experiencing violence from an intimate partner.

### Policy and public health implications

The findings of this study are relevant to policy and public health. Our findings that women from the richest households are less likely to experience IPV in their lifetime draw the attention of policymakers and key stakeholders in policy formulation and implementation to have pro-poor policies on IPV. Emphasis must be placed on the poor’s special needs to empower them and eliminate the macro-level factors like poverty that permeate IPV perpetration. This economic empowerment should be in isolation but married with gender empowerment to increase the effectiveness of IPV prevention interventions. Public health-wise, the findings identified the hotspots of IPV and mapped out the spatial distribution of IPV. This should provide public health providers with the blueprint that will guide their programs and interventions. Furthermore, it will help to ensure optimal utilisation of scarce resources in the fight against IPV in South Africa, as priority will be given to provinces that are hotspots for IPV.

### Strengths and limitations

Our study has several strengths. First, the use of nationally representative data boosts the capacity of our findings to be generalised to women in South Africa. Our study identified the hotspots of IPV in South Africa, which is a major contribution to web of literature on IPV in South Africa, as these would be beneficial to program designers and implementers in their design of context-specific and population-targeted interventions to alleviate IPV. Nevertheless, the study was not without some limitations. A major limitation of this study was that the data used was cross-sectional in design, limiting us from establishing causality. Also, the data was self-reported and therefore made it highly susceptible to recall bias, and social desirability bias since IPV is not socially acceptable in South Africa. Additionally, the data were collected 6 years ago and might not necessarily depict the current situation of IPV in South Africa.

## Conclusion

We identified Western Cape, Free State, and Eastern Cape as the hotspots for IPV in South Africa. Furthermore, women who were previously married and cohabiting were more likely to experience lifetime IPV, while belonging to the richest household, being married, and living in Limpopo, Gauteng, Mpumalanga, and North West provinces were associated with lower likelihoods of experiencing IPV. Much effort must be committed by government and non-governmental organisations to improve women’s economic status to reduce or eliminate the risk of experiencing IPV. Future studies could investigate drivers of IPV in the identified hotspots for effective and specific planning and programming.
